# Functional evaluation in orthodontic surgical treatment: long-term stability and predictability

**DOI:** 10.1186/s40510-015-0097-6

**Published:** 2015-09-21

**Authors:** Giampietro Farronato, Lucia Giannini, Guido Galbiati, Santo Andrea Stabilini, Michele Sarcina, Cinzia Maspero

**Affiliations:** Maxillo-Facial and Odontostomatology Unit (Head: prof. AB. Giannì), Fondazione Cà Granda IRCCS Ospedale Maggiore Policlinico, Milan, Italy

## Abstract

**Background:**

The introduction of electromyographic and kinesiographic technology in orthodontics allows to obtain objective data regarding the functional aspects of the mandibular movements and the masticatory muscular activity. It is then important to be able to correlate the data obtained by instrumental activity with the clinical ones. The aim of this study consists to analyse the post ortodontic surgical stability through instrumental evaluation of the masticatory muscles and mandibular movements.

**Method:**

30 patients undergo electromyographic and kinesiographic evaluations through all the surgical orthodontic iter and were than followed during other 4 years. JMP software was used to analyze and correlate the electromyographic and knesioographic data during treatment and during the follow up.

**Results:**

A linear correlations between some functional objective values collected from the examinations at the beginning and during therapy and the follow up one has been demonstrated.

**Conclusion:**

It is important to submit patients in surgical ortodontic treatment to instrumental analysis which can evidence how masticatory function and mandibular movements are performed. It is also important to highlight some functional values also from the beginning of the treatment because an alteration of such values can be related to a better or worse postsurgical rehabilitation.

## Background

During the last decades, the electromyographic and kinesiographic technology has allowed dentists to study the homeostatic response of the body to the physiological and pathological alterations of the stomatognathic system [1–3].

This has permitted to obtain quantitative data which consequently has improved the overall diagnostic process. In fact, such measurements can be statistically evaluated and are considered important not only during the diagnostic and therapeutic phases of the treatment but also during the follow-up [4, 5].

Post-orthodontic stability is guaranteed if the occlusion obtained is in harmony with the oral cavity functions and in particular with the activity of the masticatory muscles [6, 7].

Hence, it is essential to make a correct diagnosis and define a treatment plan which is based on the clinical and instrumental data obtained taking into account the functional aspect of the oral cavity [8–12].

In literature, many authors believe that an inappropriate functioning of the neuromuscular system can cause important malocclusions [4, 7, 10].

Nowadays, an electromyographic evaluation has become essential for the diagnostic process in patients undergoing combined surgical-orthodontic treatment as both electromyographic and kinesiographic assessments allow to diagnose neuromuscular problems. They permit to assess the benefits of the therapy and maintenance during the follow-up phase as they are important markers for a possible relapse. Furthermore, these diagnostic tools allow to evaluate the time interval required for the functional and kinesiological parameters to return to the pre-operative values [13–23].

At the end of treatment, it is essential that the muscles which are mainly involved in the masticatory movements are relaxed and painless with no spasm. If the muscles are fatigued or are in a condition of spasm, they must recruit more motor units to maintain the same function. Hence, these will register a higher electromyographic activity.

The aim of this study was to investigate, from an electromyographic and kinesiographic point of view, muscular functionality and the mandibular kinesiology of patients undergoing orthodontic-surgical treatment. Such assessment was carried out from the diagnostic phase to the end of treatment and follow-up with the aim of identifying whether there was a predictive model of correlation between such measurements.

In particular, this retrospective study focused on determining whether there was a correlation model between electromyographic values recorded after 4 years from the end of the orthodontic-surgical therapy and the measurements made at the beginning, during, and at the end of the treatment.

## Methods

### Selection of the sample and research procedure

This study involved 98 adult patients attending the Orthodontic department of the University of Milan.

All patients included in the study were diagnosed with a specific malocclusion and were hence eligible to receive orthodontic-surgical treatment.

Therefore, the patients have been followed from the electromyographic and kinesiographic point of view during all the therapeutic orthodontic-surgical phases starting from the diagnosis to follow-up (follow-up average = 4.3 ± 0.1 years after the end of the therapy).

Out of 98 adult patients, 30 have been selected, 12 males and 18 females, aging between 19 and 54 years old (average age 31.18 ± 7.63).

For the selection of the 30 patients, the inclusion criteria were as follows:

- Adult age (≥18 years).
- Presence of a dentoskeletal discrepancy and the need for combined surgical orthodontic treatment. The choice of surgical treatment was related to every single case. Some of them were bimaxillary surgical operations, and others involved only one maxillary bone.
- Electromyographic and kinesiographic exams during the following stages:
  - Diagnosis (to obtain additional information to the clinical and radiographic methods evaluations)
  - Before bonding
  - Every 2 months during the pre-surgical orthodontic phase and monthly immediately before and after surgery
  - The day before surgery
  - Before the intermaxillary fixation phase
  - After the intermaxillary fixation phase
  - During the post-surgical orthodontic phase
  - At the removal of the surgical bite
  - At the de-bonding phase
  - In follow-up controls
- Presence of a post-treatment follow-up of at least 4 years.

Patients who did not satisfy all inclusion criteria were excluded.

Data collection involved a questionnaire which included information on age, weight, job, sex, previous orthodontic treatment, and temporo-mandibular disorders.

The assessments were performed by a single operator in order to eliminate operator variability.

No ethical approval or ethical review board judgment was necessary because the electromyographic tests were not invasive and represent an important part of the diagnostic phase.

### Equipment

The de Gotzen electromyograph Freely and Myotronics electromyography and kinesiography K6-I was used on all patients.

Each patient underwent an electromyographic and a kinesiographic exam performed with the K6-I equipment during all phases of the orthodontic-surgical treatment and in particular during the diagnostic phase, at the bonding, during the pre-surgical treatment, the day before surgery, during the intermaxillary fixation period, at the de-bonding, and in the follow-up phases.

### Analysis and data interpretation

The statistical analysis of this study has been performed using JMP statistical discovery software (2010).

This program allows to calculate correlation predictive models through multiple linear regression methods in which a series of data known as dependent variables (which we have referred to as fundamental measure) is related to a series of independent variables.

The aim was to highlight a significant correlation between the electromyographic values obtained at 4 years after the orthodontic-surgical treatment and the values obtained at the beginning and during the therapy.

The JMP software is able to establish a correlation between the electromyographic evaluation undertaken after 4 years of the end of treatment and the previous evaluations made at the beginning, during, and at the end of treatment.

Concerning the electromyographic index obtained from the exams made before and during treatment, the following parameters have been assessed:

- The TORS at the beginning of treatment
- The value expressed in micronvolts of the right masseter muscle activity in maximum voluntary clench (MVC) at the beginning of treatment
- The value expressed in micronvolts of the left masseter muscle activity in MVC at the beginning of treatment
- The value expressed in micronvolts of the anterior left temporal muscle activity in MVC at the beginning of treatment
- The value expressed in micronvolts of the anterior right temporal muscle activity in MVC at the beginning of treatment
- The percentage overlapping coefficient (POC) value at the beginning of treatment
- The asymmetry value at the beginning of treatment
- The activation value at the beginning of treatment
- The relaxing percentage of the muscle pairs after transcutaneous electrical neuromuscular stimulation (TENS) at the beginning of treatment
- The value expressed in micronvolts of the right masseter muscle activity at rest during the intermaxillary block
- The value expressed in micronvolts of the left masseter muscle activity at rest during the intermaxillary block
- The value expressed in micronvolts of the anterior left temporal muscle activity at rest during the intermaxillary block
- The value expressed in micronvolts of the anterior right temporal muscle activity at rest during the intermaxillary block
- The steepness of the recovery curve after surgery
- The age
- The sex
- The weight

In addition, the following information was included:

- Previous orthodontic treatment
- Temporo-mandibular disorders
- The skeletal class
- The type of surgery (maxillary, mandibular, and maxillary and mandibular ones)

Through the distance method of Mahalanobis, we have identified and excluded the outlier values which must be excluded in the evaluation of the linear regression model.

Once the outlier values were eliminated, it was necessary to identify which of the independent variables taken as reference in the sample was to be included in the model. To do this, the linear correlation coefficient between the selected couple of dependent and independent variables was assessed.

In case of two variables, the linear correlation coefficient is given by the ratio between the co-variance and the product of the corresponding deviations. This coefficient ranges between −1 and +1.

Where the coefficient is equal to +1, there is a directly proportional positive linear correlation.

Where the coefficient is equal to −1, there is a directly proportional negative linear correlation.

If the coefficient is equal to 0, there is no correlation.

The JMP software allows to evaluate the linear correlation coefficient between the couple of variables identified.

In cases where the coefficient was equal to 0 between an independent variable and a dependent one, the assumption was that for the dependent value there was no correlation and hence it was to be excluded from the model.

If the linear correlation coefficient between two dependent variables was equal to +1, we assumed that there was binding co-linearity between the two dependent variables. In such cases, only one of the two was included in the model.

Regression analysis is a technique of multivariate statistical analysis which aims at identifying the relationship between a variable (dependent var iable) and the ensemble of explanatory variables (independent variables) through a correlation model obtained by a linear regression procedure. A *t*-test for each independent variable is performed to verify if the parameter *b* is different from 0 (i.e. if there is a statistically significant correlation).

Thereby, if the test was significant, there was a linear dependence between the dependent variable and the independent ones selected. Therefore, all independent variables, for which the test was not significant, were removed. The Fisher test was then applied which allows to check if the number of independent variables is sufficient to describe the dependent variable.

The variables in which the test was less significant were removed.

If one or more variables were not significant, the less significant variable was eliminated. The model was then simplified, and the procedure was repeated until all variables were maintained.

## Results

From the analysis of the clinical cases considered, 16 independent variables were selected to correlate with the fundamental measure. For each of the variables, the distribution has been displayed both graphically and statistically through the evaluation of the quantiles, average, and standard deviation.

**Fig. 1:**
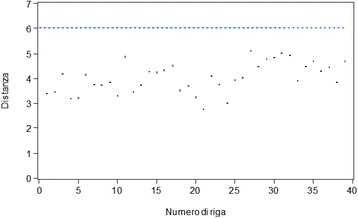
Mahalanobis distances

The distribution analysis allows to exclude the presence of an anomalous value. According to the Mahalanobis distances method, the results of which are shown in Fig. 1, it was highlighted that all the data was below the critical value.

None of the data included in the study reach the critical value established by the Mahalanobis technique; hence, all patient data were acceptable.

**Fig. 2:**
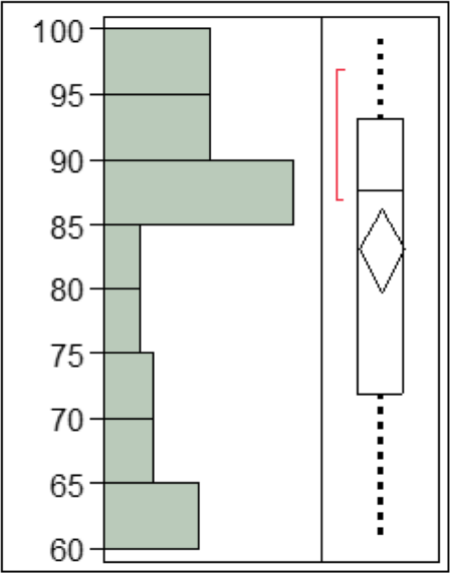
Fundamental measure expressed in percentage

**Fig. 3:**
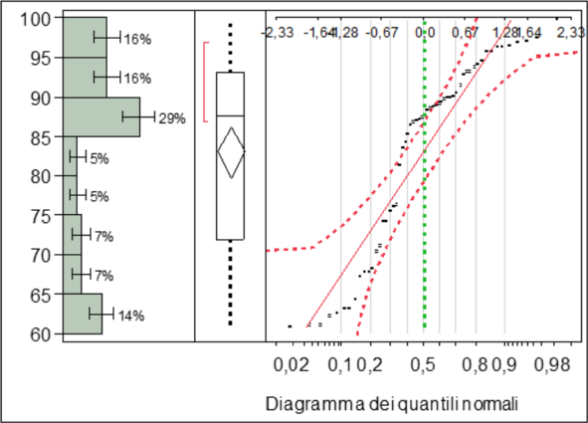
Normal quantile diagram of the fundamental measure

Analyzing graphically the distribution of the fundamental measure and the independent variables, the following were observed:

- The fundamental measure ranges from a minimum of 60.95 to a maximum of 99.75 with an average value of 83.13. Such value is considered optimal.
- The data concerning the fundamental measure was distributed randomly with a high number of patients with a fundamental measure between 85 and 90.
- In relation to the age, the variable was between 19 and 54 years with an average age of 30.98.
- Only one case over the age of 50 was registered
- The mean age was between 25 and 30 years old (Figs. 2 and 3).

After isolating the patients that had a fundamental measure between 85 and 90 %, we noted the following:

- They were not represented by a precise age.
- They were not represented by a specific body weight.
- They showed an initial torsion index (TORS) value lower than 10 %, therefore, considered standard.
- They had an initial percentage overlapping coefficient (POC) value higher than 75 %.
- They had the masseter muscle values in micronvolts at the beginning of treatment higher than 400 μV.
- They had the temporal muscle values in micronvolts at the beginning of treatment higher than 200 μV.
- They had an asymmetrical value between −10 and 10 %, considered physiological.
- They had an activation value between −10 and 10 %, considered physiological.
- Relaxing after TENS was lower than 40 %, reaching the 99 % value.
- The steepness curve is higher than 50 %.

By isolating the patients who had a fundamental measure higher than 95 %, the following were noted:

- There was no specific age.
- There was no specific body weight.
- The initial TORS value was lower than 10 %, hence, considered standard.
- The initial POC value was between 75 and 85 %.
- The masseter muscle value in micronvolts obtained at the beginning of treatment was higher than 400 μV.
- The temporal muscle value in micronvolts obtained at the beginning of treatment was higher than 200 μV.
- The asymmetrical value obtained was between −10 and 15 %, which is considered physiological.
- The activation value obtained was between −10 % and 10 %, which is considered physiological.
- The relaxation value after TENS was lower than 40 %.
- The steepness curve was higher than 50 %.

Such findings were also highlighted by the scatter graph and from the matrix of the scatter graph. Particularly, high values of the fundamental measure corresponded to the following:

- A low value of the initial TORS compared to the total.
- A high value of the temporal and masseter muscle contraction at the beginning of treatment compared to the total.
- A high value of the masseter muscle contraction at the beginning of treatment compared to the total.
- A lower and higher value compared to the standard values for what concerned the asymmetrical and activation index.
- A high value of the initial POC.
- A low value of the masseter and temporal muscles during the intermaxillary block.
- A high value of the steepness curve and of the relaxing value after TENS.
- There were no correlations with weight, age, and skeletal class.

**Fig. 4:**
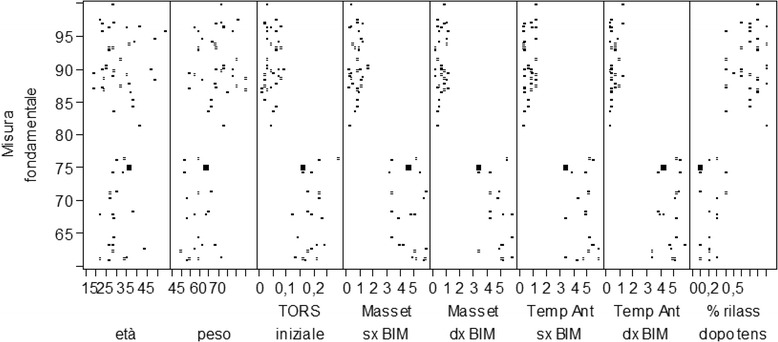
Release matrix graphic ABOVE

**Fig. 5:**
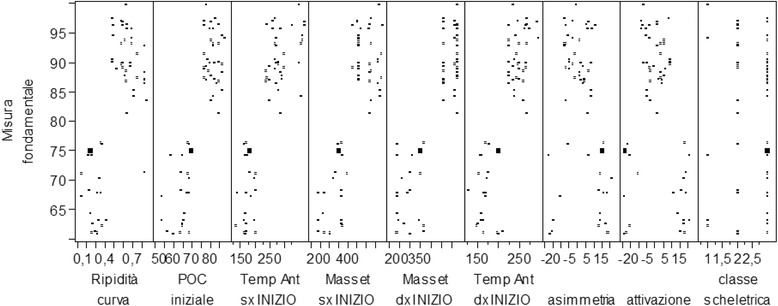
Release matrix graphic BELOW

Instead, the following corresponded to the low values of the fundamental measure:

- A high value of the initial TORS compared to the total.
- A low value of the temporal and masseter muscles contraction at the beginning of treatment compared to the total.
- A low value of the masseter muscle contraction at the beginning of treatment compared to the total.
- Within the standard values for what concerns the asymmetrical and activation index.
- A low value of the initial POC.
- A high value of the masseter and temporal muscles during the intermaxillary block.
- A low value of the steepness curve and muscle relaxation after TENS.
- There were no correlations with weight, age, and skeletal class (Figs. 4 and 5).

From the analysis of the correlation chart in which are listed the linear correlation coefficients, it was noted that the relationship between fundamental measure and age, weight, asymmetry, and activation was weak. A strong and positive relationship was observed between the relaxation percentages after TENS, the steepness of the post-surgery rehabilitation curve, and the initial POC for the values in micronvolts of the right and left temporal at the beginning of treatment and the right and left masseter at the beginning of treatment.

The relationship between the fundamental measure and initial TORS, the right and left masseter at rest during the intermaxillary block, and the right and left temporal during the intermaxillary block was strong and negative.

**Fig. 6:**
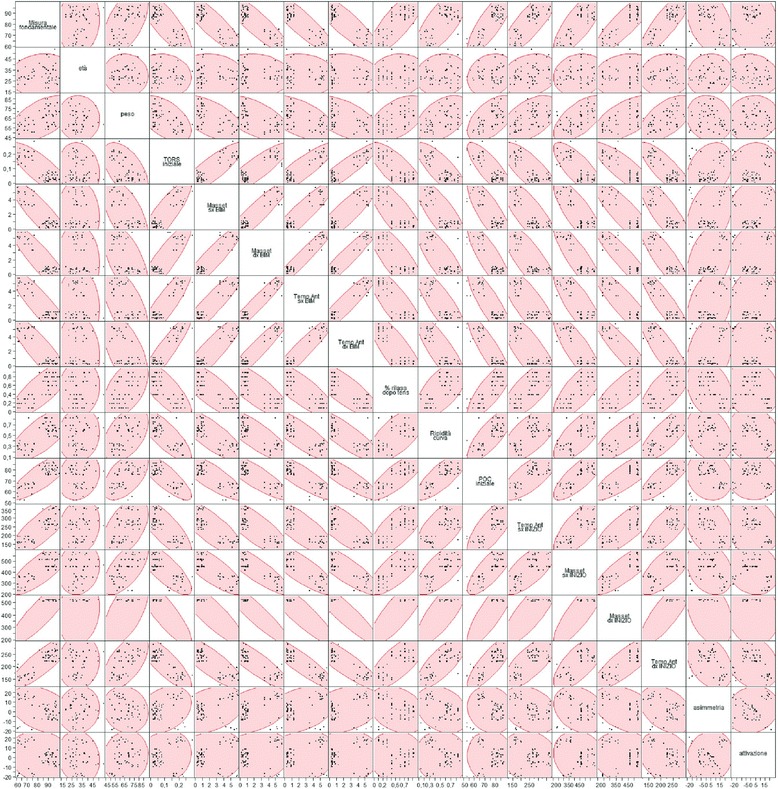
Matrix of correlations

The same type of relationship is shown in the chart in which we have reported the data through a dispersion contained in the correlation ellipses. If the ellipses are flattened and facing downwards, the correlation is strong and negative, whilst if they are flattened and facing upwards, the correlation is strong and positive. In cases where the ellipses are close to the circle shape, there is no correlation (Fig. 6).

The correlations assessed were between the fundamental measure and the following:

- Weight and age
- Asymmetry and activation
- POC and TORS at the beginning of treatment
- Right and left masseter in MVC at the beginning of treatment
- Right and left temporal in MVC at the beginning of treatment
- Right and left temporal at rest during the intermaxillary block
- Right and left masseter at rest during the intermaxillary block
- Relaxing post-TENS and curve steepness
- Skeletal class and surgery
- Previous orthodontic treatment and temporo-mandibular disorders

**Fig. 7:**
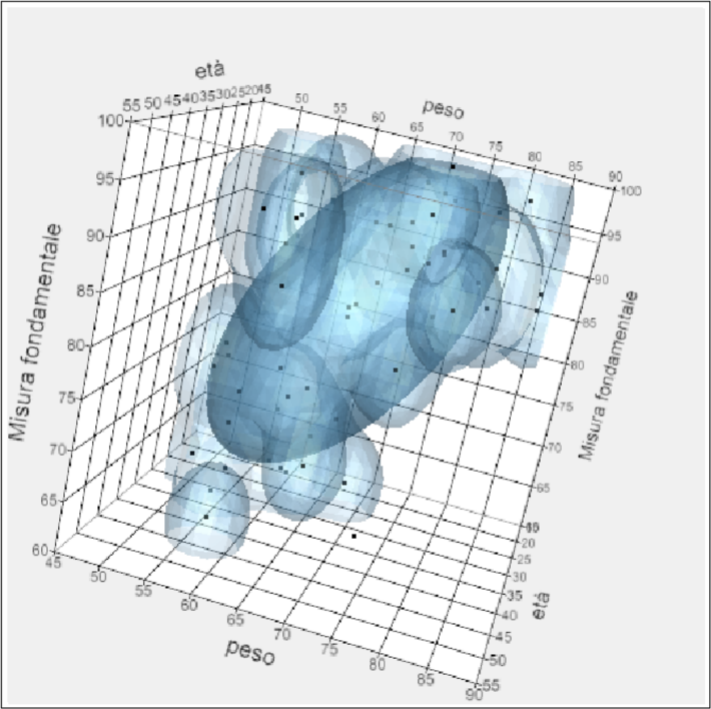
Correlation between fundamental measure, age, and weight

**Fig. 8:**
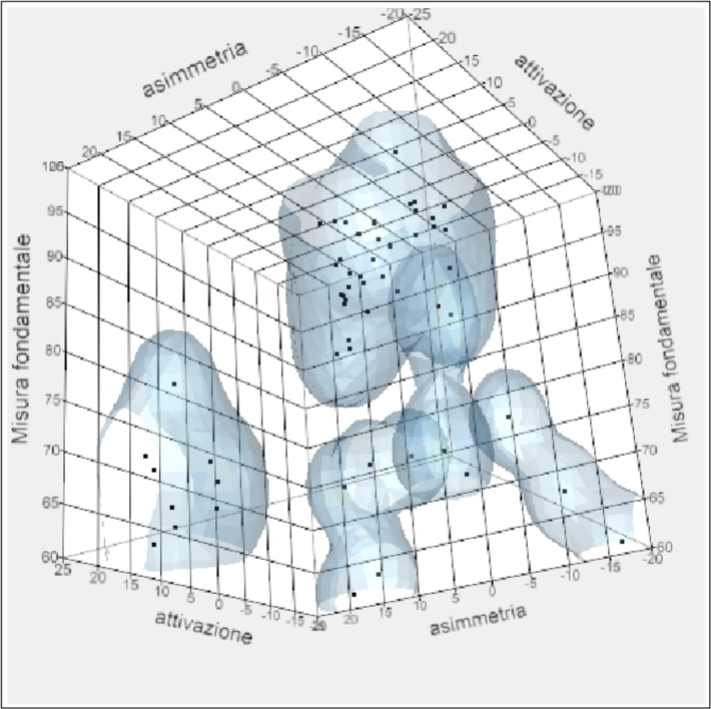
Correlation between fundamental measure, asymmetry, and activation

There was no correlation between the fundamental measure and age-weight and asymmetry activation (Figs. 7 and 8).

From the chart, a correlation between the initial POC and TORS and the fundamental measure is observed. In addition, from the chart, it is highlighted that as the initial TORS index increases, the value of the fundamental measure decreases, and as the initial POC index increases, the fundamental value increases.

**Fig. 9:**
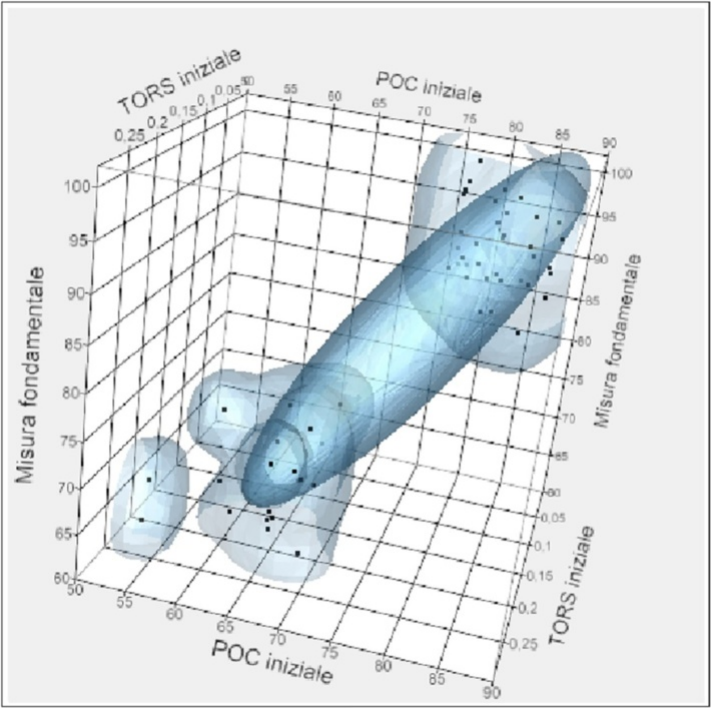
Correlation between fundamental measure and initial POC and TORS

Therefore, for the POC index, there is a positive type of correlation, whilst for the TORS index, the correlation is negative (Fig. 9).

Regarding the correlation between the value expressed in micronvolts of the right and left masseter in MVC at the beginning of treatment, it is noticeable that there is a positive correlation. In fact, with the increase of the masseter muscle activity, the value of the fundamental measure increases.

**Fig. 10:**
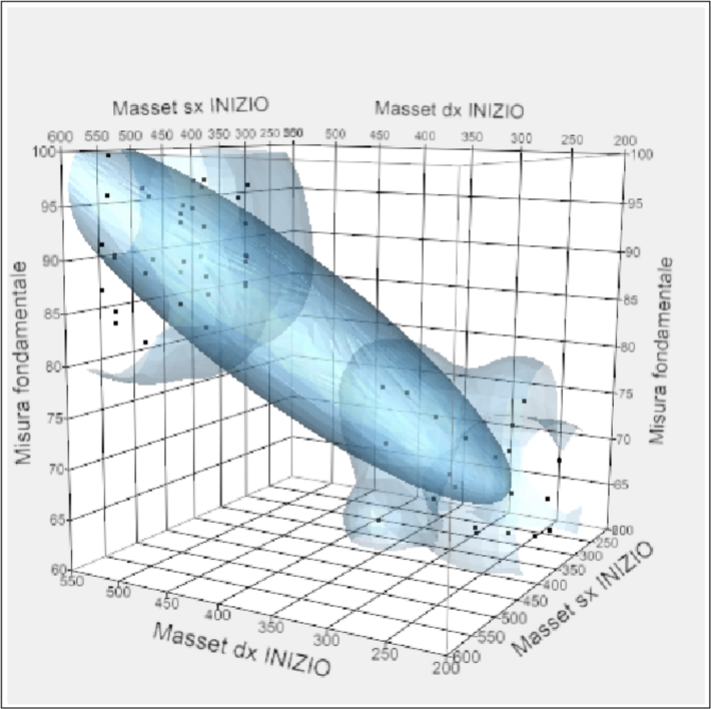
Correlation between fundamental measure and the masseter values expressed in micronvolts at the beginning of treatment

Therefore, patients who have a high masseter muscle activity at the beginning of treatment will probably have a high fundamental measure after a long period of time (Fig. 10).

**Fig. 11:**
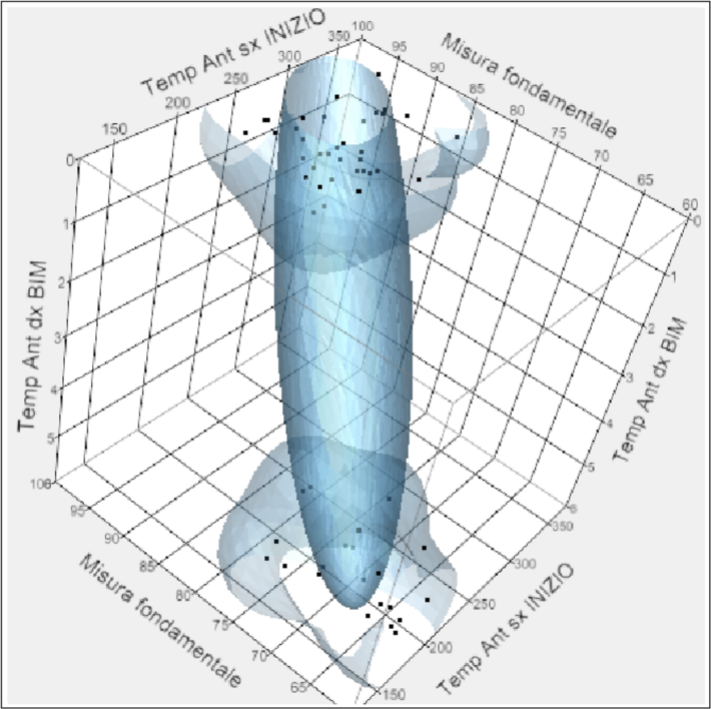
Correlation between fundamental measure and the temporal values expressed in micronvolts at the beginning of treatment

Concerning the correlation between the value expressed in micronvolts of the right and left temporal in MVC at the beginning of treatment and the fundamental measure, a positive correlation is noticeable. In fact, as the activity of the temporal muscle increases, the fundamental value increases. Hence, patients who have a high activity of the temporal muscles at the beginning of treatment will have a high fundamental measure after a long period of time (Fig. 11).

Analyzing the correlation model between the value expressed in micronvolts of the right and left masseter muscle at rest during the intermaxillary block and the fundamental measure, a negative correlation is noticeable (when this value increases, the fundamental measure decreases). In fact, patients who have a high masseter muscle activity at rest during the intermaxillary block have a low fundamental measure at the long term follow-up.

**Fig. 12:**
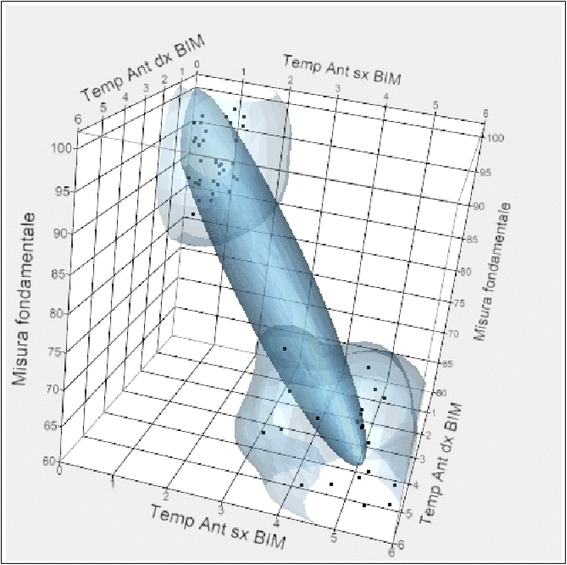
Correlation between fundamental measure and the temporal values expressed in micronvolts during the intermaxillary block

Analyzing the correlation model between the value expressed in micronvolts of the right and left temporal muscle at rest during the intermaxillary block and the fundamental measure, a negative correlation is noticeable; hence, when this value increases, the fundamental measure decreases (Fig. 12).

The relationship between the fundamental measure and the percentage of muscle relaxation post-TENS proved a positive correlation, i.e., the higher is the muscular relaxation post-TENS, the higher is the level of the fundamental measure at the long-term follow-up.

**Fig. 13:**
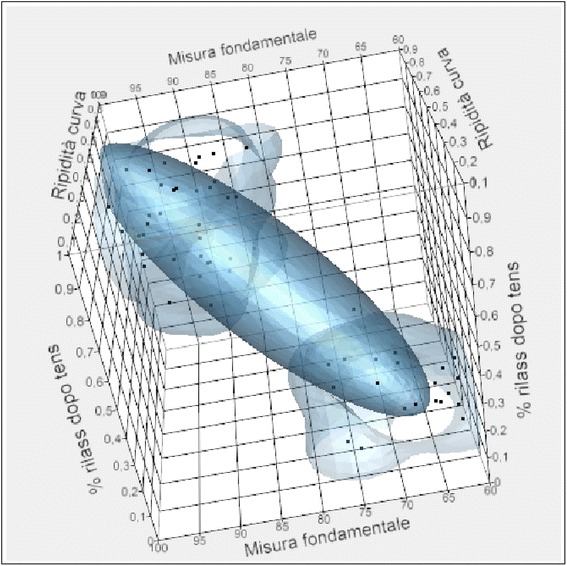
Correlation between fundamental measure and relaxing percentage post-TENS and the curve steepness

There also is a positive correlation between the steepness curve and the fundamental measure; hence, the steeper is the post-surgery rehabilitation curve, the higher is the fundamental measure. This implies that patients who have a fast neuro-muscular post-surgery recovery will have the best electromyographic values at the long-term follow-up (Fig. 13).

Once the correlations were identified, we proceeded to the evaluation of the multivariate linear correlation model using the 16 independent variables. The variables for which the evaluation of the estimation parameter was not equal to the student test were eliminated.

It was assumed that in the fundamental hypothesis of the test (H), the correlation parameter was 0, i.e., there was no correlation. If the *t* value was higher than the limit value evaluated with a significance threshold of 5 %, we rejected the H hypothesis and hence we considered it as a significant correlation. Once the variables were removed, the model remained with nine variables which all passed the student test.

Once the *p* value concerning Fisher’s *F* test was lower than 0.0001, Fisher’s test was passed. Consequently, the variables were sufficient to describe the relationship between the fundamental measure and the independent variables selected. The graph shows the comparison between the values of the fundamental measure expected and the ones observed. It is highlighted that all values are found on a 45° straight line.

This data proves that the values expected by the model are close to the actual observed values.

In particular, the value of the *R* chart is high and equal to 0.871 which is considered excellent as it is close to 1.

### Complete model

**Fig. 14:**
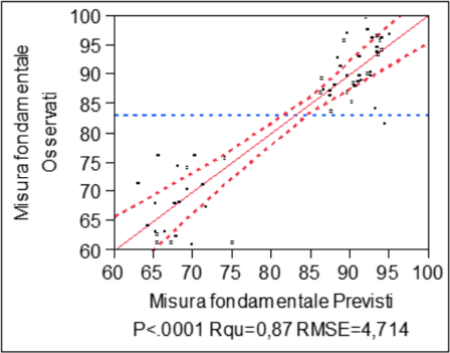
Ex graph of the observed answers compared to the expected ones

From the variance analysis, there is a Fisher distribution *p* value lower than 0.0001 (Fig. 14 and Table 1).

**Table 1:** Estimate summary

R-squared	0,871829
Correct R-squared	0,846752
Mean Square Deviatio	4,713992
Average of Results	83,13625
Weighted Addition	30

### Variance analysis

The *p* value of the Student *t*-test distribution was lower than 5 % (Tables 2, 3, and 4).

**Table 2:** Variance analysis

Origin	DF	Addition of squares	Quadratic means	F ratio
Model	9	6953,0949	772,566	34,7663
Error	46	1022,1992	22,222	Prob > F
C. total	55	7975,2941		<,0001*

**Table 3:** Missed estimate

Origin	DF	Addition of squares	Quadratic means	F ratio
Miss Evaluation	45	1022,1984	22,7155	28394,40
Neat Error	1	0,0008	0,0008	Prob > F
Total Error	46	1022,1992		0,0047*
				Max. r-square
				1,0000

**Table 4:** Estimates and parameters

Term	Evaluation	Std error	T - error	Prob>|t|
Intercept	54,118739	14,92805	3,63	0,0007*
Initial TORS	14,607324	22,73505	0,64	0,0005*
Temp Ant sx BIM	−1,493326	1,248338	−1,20	0,0009*
Temp Ant dx BIM	−1,149171	1,382826	−0,83	0,0003*
% relax after tens	3,8688461	4,69093	0,82	0,0002*
Rake curve	−8,1938	6,524735	−1,26	0,0007*
INITIAL Temp Ant sx	0,0328983	0,018219	1,81	0,0004*
INITIAL Masset sx	0,0080032	0,013443	0,60	0,0007*
INITIAL Masset dx	0,0212825	0,017359	1,23	0,0007*
INITIAL Temp Ant dx	0,0658332	0,035948	1,83	0,0004*

**Fig. 15:**
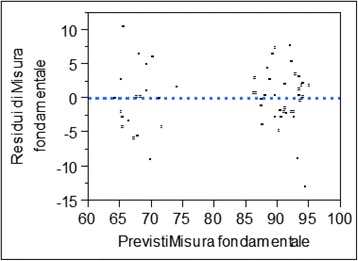
Residual

From the residual analysis, it is noticeable that they are uniformly distributed around 0 which shows how the linear correlation hypothesis is acceptable (Fig. 15).

In the graphs are reported the leverage coefficient values for each of the dependent variables.

The leverage coefficient (stimulus, influence) that varies between 0 and 1 is a measure of how much a given value of the independent variable deviates from its average.

Finally, we deduced the prevision model.

### Prevision expression

- Initial TORS
- Left temp intermaxillary fixation (BIM)
- Right temp BIM
- Relaxing % after TENS
- Steepness curve
- Left temp beginning
- Left masseter beginning
- Right masseter beginning
- Right temp beginning

From this formula, it is highlighted that some values for which a previous correlation had been shown proved to be not statistically significant.

Statistically significant correlations have been proved between the fundamental measure and the following:

- The TORS at the beginning of treatment
- The values expressed in micronvolts of the temporal and masseter muscles in MVC at the beginning of treatment
- The relaxing percentage after TENS
- The temporal muscle values at rest during the intermaxillary block
- The steepness value of the rehabilitation curve in the post-surgical phase

Therefore, the values of the masseter muscles expressed in micronvolts during the intermaxillary block and the POC initial value are not correlated to the fundamental measure in a statistically significant way.

To give more authenticity to the correlations obtained, we assumed to have no data of the fundamental measure and tried to deduce them from the previous electromyographies. Hence, we tried to predict the electromyography values at the follow-up on the basis of the previous data obtained.

From the graph of the observed values (actually registered) and the expected ones, it is highlighted how the model is able to foresee the data at both high values and low value of the fundamental measure.

**Fig. 16:**
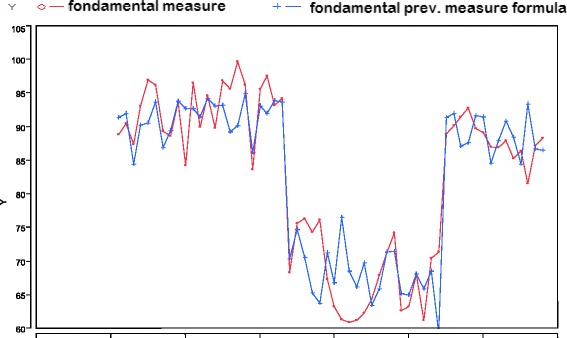
Overlaid graph between expected and observed data

**Fig. 17:**
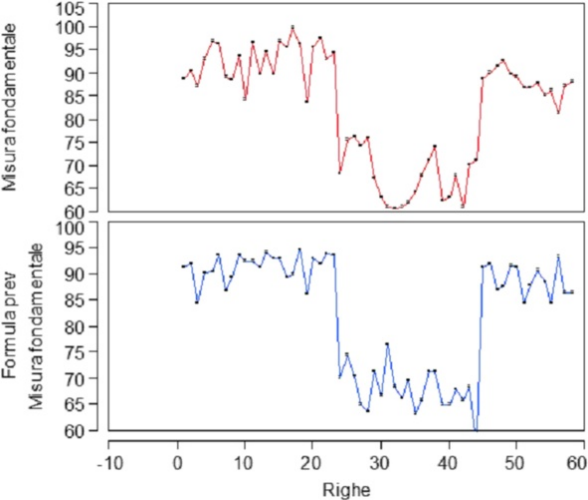
Overlaid graph between expected and observed data divided in two diagrams

This allows to deduce that the model is predictive (Figs. 16 and 17).

## Discussion

The data collected and analyzed in this study allowed to reach a few conclusions on the functional post-surgery rehabilitation in patients receiving combined surgical-orthodontic treatment and concerning the presence of correlations between the patients’ neuromuscular situation at the beginning of treatment and in the follow-up [24–37].

It was possible, in fact, to deduce that patients receiving surgical-orthodontic treatment had indices that allowed to formulate a prognosis, i.e., to estimate the neuromuscular function and measure at the follow-up.

From a first data analysis, we considered all electromyographic and kinesiographic values collected from the diagnostic phase to the follow-up [38, 39].

The values that were not statistically significantly correlated between themselves have been rejected. In particular, the kinesiographic values have been rejected both because they were not significantly correlated and because it is known from the studies of Tate et al. and Throckmorton et al. that the long-term kinesiographic rehabilitation is not so quick and satisfying as the electromyographic one [24, 25, 34].

By selecting the data, we have obtained 16 indexes, or independent variables, obtained from the electromyographies made before and during treatment. Among these, some data was specific to the patient (weight and age), some referred to the electromyographies at the beginning of treatment (initial POC and TORS, values expressed in micronvolts for the four muscles at the beginning of treatment, asymmetry, and activation values), other data referred to the pre-surgical phase (relaxing % after TENS), and others referred to the intermaxillary block phase (values at rest for the four muscles) and the post-surgical phase (steepness of the rehabilitation curve in the post-surgical period).

From the distribution’s analysis, we noted that patients with high, medium, or low fundamental measure values had specific characteristics both at the beginning and during treatment. The statistical model utilized allowed the elimination of values which did not correlate (backward elimination). Therefore, the values less correlated were the first ones to be excluded until only highly correlated values were obtained.

These last values obtained are then subjected to further statistical analysis which allows to find the values which correlate to the fundamental measure.

This correlation model allows, at the end of treatment, to obtain indices statistically correlated to the fundamental measure. In this study, these indices were the initial TORS, the values expressed in micronvolts for the four muscles at the beginning of treatment, the temporal muscle values during the intermaxillary block, the relaxing percentage after TENS, and the steepness of the curve during post-surgery.

This model can be useful to estimate, during the treatment phase, the post-surgical rehabilitation level of the patient.

In literature, there are no studies that show the same correlation. One study in 2011 [39] had shown that high values of the temporals’ activity at rest during the intermaxillary block were a negative prognostic index.

## Conclusions

It is important that the combined surgical-orthodontic treatment is planned taking into consideration the functional needs. The surgical repositioning of the bone bases determines alterations of both the anatomy and function and of the relationship of the various anatomical structures. Hence, a functional evaluation is essential [40–46].

This study highlights the importance of monitoring patients in the long term, especially several years after the end of treatment [47–50].

In literature, many authors have published works similar to this; however, there are still many doubts and uncertainties.

In particular, there is no evidence on studies that have correlated the values obtained at the end of treatment with those collected during treatment.

In conclusion, each patient responds to treatment differently, and therefore, there is a big individual variability in terms of neuromuscular and functional response to the orthodontic-surgical therapy. However, it is possible to make correlations between electromyographic evaluations made at the follow-up and those made before and during treatment. In fact, we have reached a linear correlation between the electromyographic values obtained before and during therapy and those registered at the follow-up which could allow to make predictive evaluations of the neuromuscular stability in the long term.
